# Incongruence between dominant commensal donor microbes in recipient feces post fecal transplant and response to anti-PD-1 immunotherapy

**DOI:** 10.1186/s12866-021-02312-0

**Published:** 2021-09-20

**Authors:** Hyunmin Koo, Casey D. Morrow

**Affiliations:** 1 Department of Genetics Hugh Kaul Personalized Medicine Institute, University of Alabama at Birmingham, Alabama Birmingham, USA; 2 Department of Cell, Developmental and Integrative Biology Hugh Kaul Personalized Medicine Institute, University of Alabama at Birmingham, Alabama Birmingham, USA

**Keywords:** Fecal microbe transplantation, Anti-PD-1 immunotherapy, Metagenomics, Strain tracking, GI tract colonization

## Abstract

**Background:**

To understand inter-individual variability of fecal microbe transplantation (FMT) to enhance anti-PD-1 immunotherapy (IT) for melanoma, we analyzed the data sets from two recent publications with a microbial strain-tracking tool to determine if donor strains were dominant in the recipient feces following FMT.

**Results:**

Analysis of the Baruch et al. data set found that the presence of commensal donor microbes in recipient feces post-FMT did not correlate with the patient response to IT. From the Davar et al., data set, we found 4 patients that responded to IT had donor’s related strain post-FMT, while 2 patients that did not respond to the IT also had donor’s strain post-FMT. Importantly, we identified no donor microbes in the feces in one recipient post-FMT that responded to IT. Furthermore, in depth analysis from two patients who responded to IT revealed both donor and recipient strains at different times post-FMT. Colonization of the gastrointestinal tract niches is important for the interaction with the host immune system. Using a separate data set, we show that mucosa from the cecum, transverse colon, and sigmoid colon share strains, providing a large reservoir of niches containing recipient microbes.

**Conclusions:**

We demonstrated using strain-tracking analysis individual variation with the respect to the presence of fecal dominant donor microbes in the recipient following FMT that did not correlate with the response to anti-PD-1 immunotherapy. The inter-individual differences of FMT to enhance IT might be explained by the variability of the donor microbes to occupy and outcompete recipient microbes for the gastrointestinal niches. The result from our study supports the use of new approaches to clear the niches in the gastrointestinal tract to promote donor colonization to reduce inter-individual variability of IT for melanoma and potentially other cancers.

**Supplementary Information:**

The online version contains supplementary material available at 10.1186/s12866-021-02312-0.

## Background

The composition of the gut microbial community has been shown to be a regulator of the response to anti-PD-1 immunotherapy (IT) for a variety of cancers [1–4]. Recently, several studies have reported that fecal microbe transplantation (FMT) enhanced IT for melanoma in humans [5, 6]. Interestingly, in both studies, patients were given FMT with feces from individuals that had responded to IT. However, the FMT in these individuals had varied success and, in some instances, the same donor enhanced survival in some patients but not in others [5, 6].

Most of the clinical success for FMT to improve or alleviate symptoms has been found when given to relieve patient’s chronic recurrent *Clostridium difficile* [7–10]. Subsequent studies reported sporadic success using FMT for the treatment of diseases such as obesity, diabetes, IBD, and Crohn’s disease[7]. To further understand the mechanism of FMT, later studies have used an analysis that combined metagenomic DNA sequencing with new informatics to investigate the microbial strain community [11–13]. In a previous study, we used metagenomic DNA sequencing analysis with a Window-based Single Nucleotide Variant (SNV) Similarity (WSS) program to assess the strain relatedness of the microbes in two separate samples from the same individual [11]. Using paired samples from the data set from the Human Microbiome Project (HMP), we established cut-off values for the WSS scores that can discern between related and unrelated samples [11, 14, 15]. Using the WSS strain-tracking method, we have shown that FMT in patients with recurrent *C. difficile* results in the presence of donor microbes in the feces of recipients for up to two years with no evidence of residual recipient fecal stains [11].

In the current study, we have used the WSS strain-tracking method to analyze the FMT from two studies that have used FMT to enhance IT [5, 6]. Both studies have reported the favorable outcome of FMT to enhance IT in patients with melanoma. In addition, they have provided extensive post FMT longitudinal sampling and results from metagenomic sequencing that allows for strain tracking of donor microbes in recipients post FMT. Our analysis demonstrates that the donor microbe strains in the feces of the recipient following FMT do not consistently predict a positive therapeutic response for IT. Consistent with this result, the analysis of serial fecal samples from the recipient’s post-FMT reveals temporal variation between the donor and recipient microbes for fecal dominance. To put this result in perspective, we incorporated a data set from a recent study that used a unique strategy to assess the microbial composition in the mucosa from the intestinal epithelium from the cecum, transverse colon, and sigmoid colon [16]. We have used the WSS method to show that shared microbial strains are found in each of the mucosa from the intestinal epithelium of these different segments of the gastrointestinal tract (GI) intestinal epithelium. Our analysis demonstrates the presence of the donor microbes in the feces of the recipient’s post-FMT is out of place and thus not predictive of the response to IT and supports additional approaches to increase the donor microbe occupancy of niches of the GI tract that are important for microbial interaction with the host immune system.

## Results

### Strain-tracking analysis in the feces of recipients post FMT

**Fig. 1:**
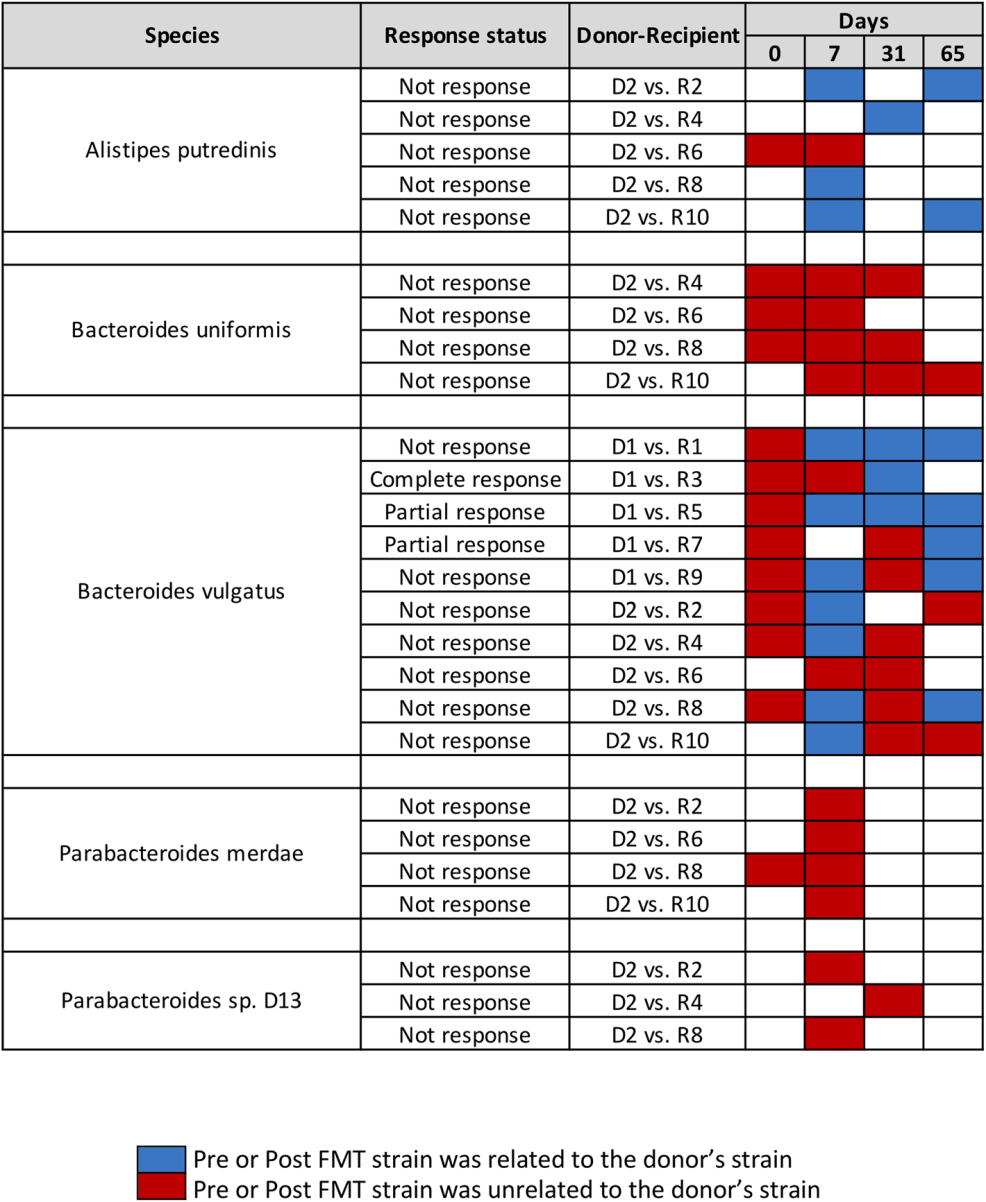
WSS scores from donor-recipient pairs from Baruch et al. The WSS scores were observed comparing the donor’s sample to paired recipient’s pre- and post-FMT samples (0, 7, 31, and 65 days). All samples used for the analysis were listed in Table S1. The WSS scores per each donor-recipient pair were grouped into different color boxes (see the figure key for detailed information). Major species found in the fecal sample and common ones observed in the two different data sets (Baruch et al. and Davar et al.) used in this study are included in this figure. The remaining species found in this data set are shown in Fig. S1, and WSS scores for all pairwise comparisons are provided in Table S2. The white boxes indicate the microbial strains that we were unable to determine relatedness due to the any sample in pairs not satisfying the criteria of WSS analysis (minimum coverage > 30 % and average depth > 3.5)

For Baruch et al., we conducted WSS strain-tracking analysis between donor-recipient pairs to determine donor strain in the feces of the paired recipient’s samples [5]. A total of 2 donors and 10 recipients (5 recipients for each donor) were used for the analysis, and we found that donor’s strain, including *Alistipes putredinis*, *Bacteroides vulgatus*, *Bifidobacterium adolescentis*, *Collinsella aerofaciens*, *Coprococcus eutactus*, *Eubacterium eligens*, *Eubacterium rectale*, *Faecalibacterium prausnitzii* A2, *Faecalibacterium prausnitzii* L2, *Prevotella copri*, and *Roseburia intestinalis* was related to the recipients’ post-FMT strain at certain time points (Fig. 1 and Fig. S1). Within these species, 6 species (*B. vulgatus, B. adolescentis, C. aerofaciens, E. rectale, F. prausnitzii* L2, and *R. intestinalis*) were showing that 36 cases had donor’s related strain in recipient’s post-FMT regardless of whether there was a clinical response or no response to IT (Fig. 1 and Fig. S1). Overall, from the WSS strain-tracking analysis, we observed that there was no correlation between FMT and response to IT as evidenced from our finding that the donor strain was found in the recipient’s post-FMT samples with either response or no response to IT. The presence of donor strains in these samples might have been due to the experimental design in which pills containing the donor feces were given multiple times during the study [5].

**Fig. 2:**
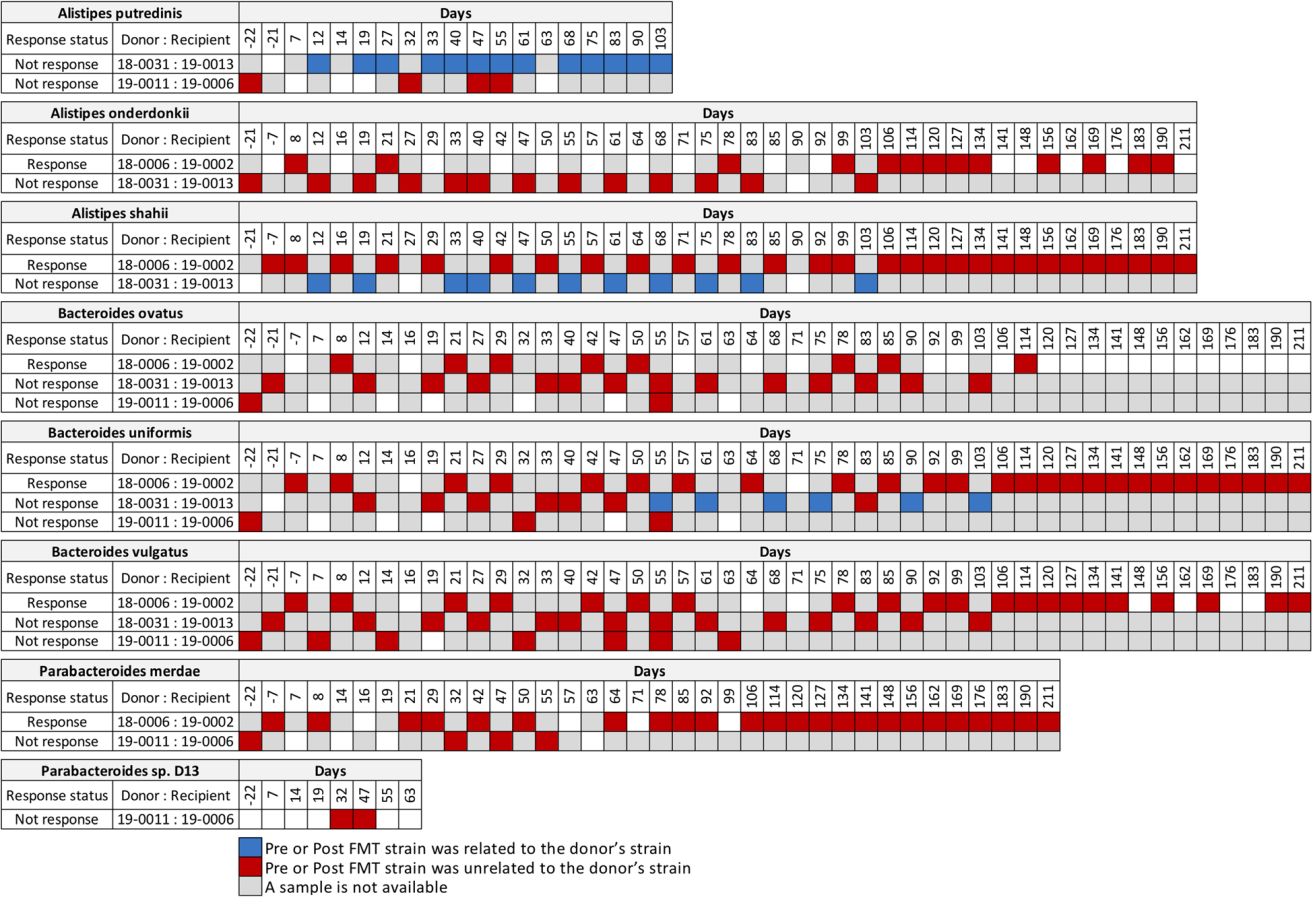
WSS scores from FMT with single donor-recipient pairs. Using the data from Davar et al., the WSS scores were determined by comparing the donor’s sample to every available paired recipient’s samples. The number of pre- and post-FMT samples from each recipient varies, and all samples used for the analysis were listed in Table S1. The WSS scores per each donor-recipient pair were grouped into different color boxes (see the figure key within the figure for detailed information). Major species found in the fecal sample and common ones observed in the two different data sets (Baruch et al. and Davar et al.) used in this study are included in this figure. The remaining species found in this data set are summarized, shown in Fig. S2, and WSS scores for all pairwise comparisons are provided in Table S3. The white boxes indicate the microbial strains that we were unable to reliably determine relatedness due to the any sample in pairs not satisfying the criteria of WSS analysis (minimum coverage > 30 % and average depth > 3.5)

We next conducted WSS strain-tracking analysis between donor-recipient pairs from Davar et al. [6]. In this study, there was only a single FMT given to the recipients. In addition, two groups of donors were used: the first group of donors (*n* = 3) used his/her fecal sample for the FMT on a single recipient while the second group of donors (*n* = 4) used his/her fecal sample in multiple recipients (Table S1). From the WSS strain-tracking analysis of the first group of each donor and paired recipient, we found that there was no strain related between donor and recipient even though recipients responded to the IT (Fig. 2 and Fig. S2). As an example, we found one donor-recipient pair where multiple donor strains (18–0031), *A. putredinis*, *(A) shahii*, *(B) uniformis*, *B. sp.* 1-1-6, *B. sp.* 2-1-16, and *B. cellulosilyticus* were found in the recipient (19 − 0013) even though this recipient did not respond to the IT (Fig. 2 and Fig. S2).

**Fig. 3:**
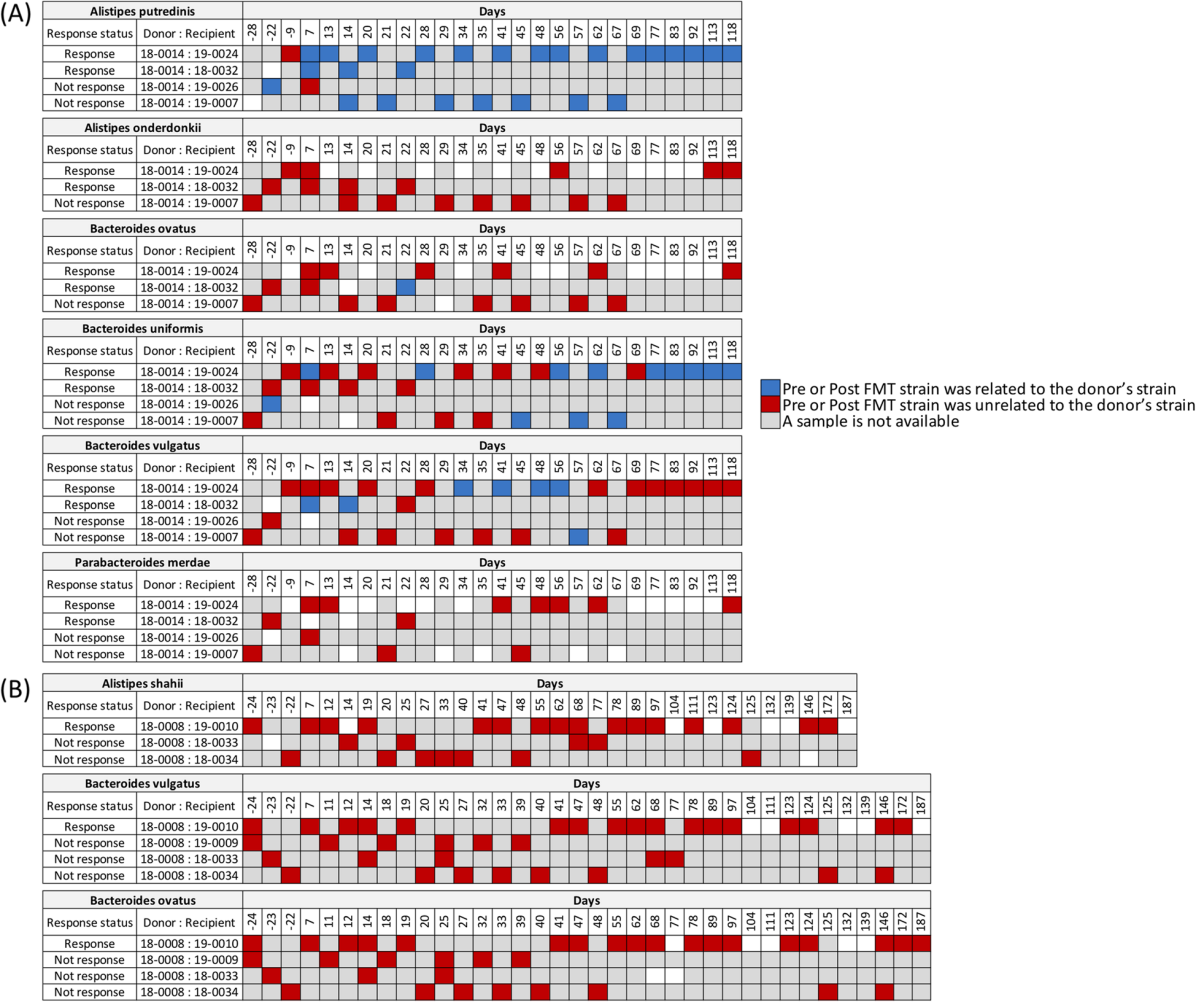
Donor microbe strains in recipients post-FMT where the same donor was used for multiple FMT. The WSS scores were determined by comparing the donor’s sample to every available paired recipients’ sample. (**A**) a donor of 18 − 0014 was used to multiple recipients (19–0026, 19–0024, 19 − 0007, 18–0032) for FMT; and (**B**) donor of 18 − 0008 was used to multiple recipients (19 − 0010, 19 − 0009, 18–0034, 18–0033). Similar to Fig. 1, the number of pre- and post-FMT samples from each recipient is varied (see Table S1 for detailed sample information). The WSS scores per each donor-recipient pair were grouped into different color boxes (see the figure key). Major species observed in the fecal sample and common ones found in the two different data sets (Baruch et al. and Davar et al.) used in this study are included in this figure. The remaining species found in this data set are displayed in Fig. S3, and WSS scores from all pairwise comparisons are provided in Table S3. The white boxes indicate the microbial strains that we were unable to determine relatedness due to the any sample in pairs not satisfying the criteria of WSS analysis (minimum coverage > 30 % and average depth > 3.5)

In the second group analysis, we used WSS strain-tracking to determine the presence of two separate donor strains (18 − 0014 and 18 − 008) in recipients post-FMT. For the first donor (18 − 0014), we analyzed 4 different recipients: 19–0024 and 18–0032 that responded to IT and 19–0026 and 19 − 0007 that did not respond to IT (Fig. 3 A and Fig. S3). All of the FMT pairs examined had donor strains in certain *Alistipes spp.*, and *Bacteroides spp.*, regardless of whether the patient responded to IT. Specifically, we found two pairs (18 − 0014 (donor) vs. 19–0026 (recipient), and 18 − 0014 (donor) vs. 19 − 0007 (recipient)) that did not respond to IT had donor’s related strain in *(A) putredinis*, *(B) uniformis*, and *B. vulgatus* (*B. vulgatus* is specific for 18 − 0014 vs. 19 − 0007 pair) (Fig. 3 A). An interesting situation was identified when a single donor (18 − 0008) was used for three separate FMT with different recipients. The transplant of 18 − 0008 into 19 − 0010 resulted in the enhancement of IT, although we did not detect donor strains for *(A) shahii, (B) vulgatus* or *B. ovatus* in the recipients at times up to 187 days post-FMT (Fig. 3B). The transplant of 18 − 0008 into the recipient 18–0033, 18–0034 or 19 − 0009 did not result in enhanced IT and we did not detect donor microbe strains after FMT (Fig. 3B).

**Fig. 4:**
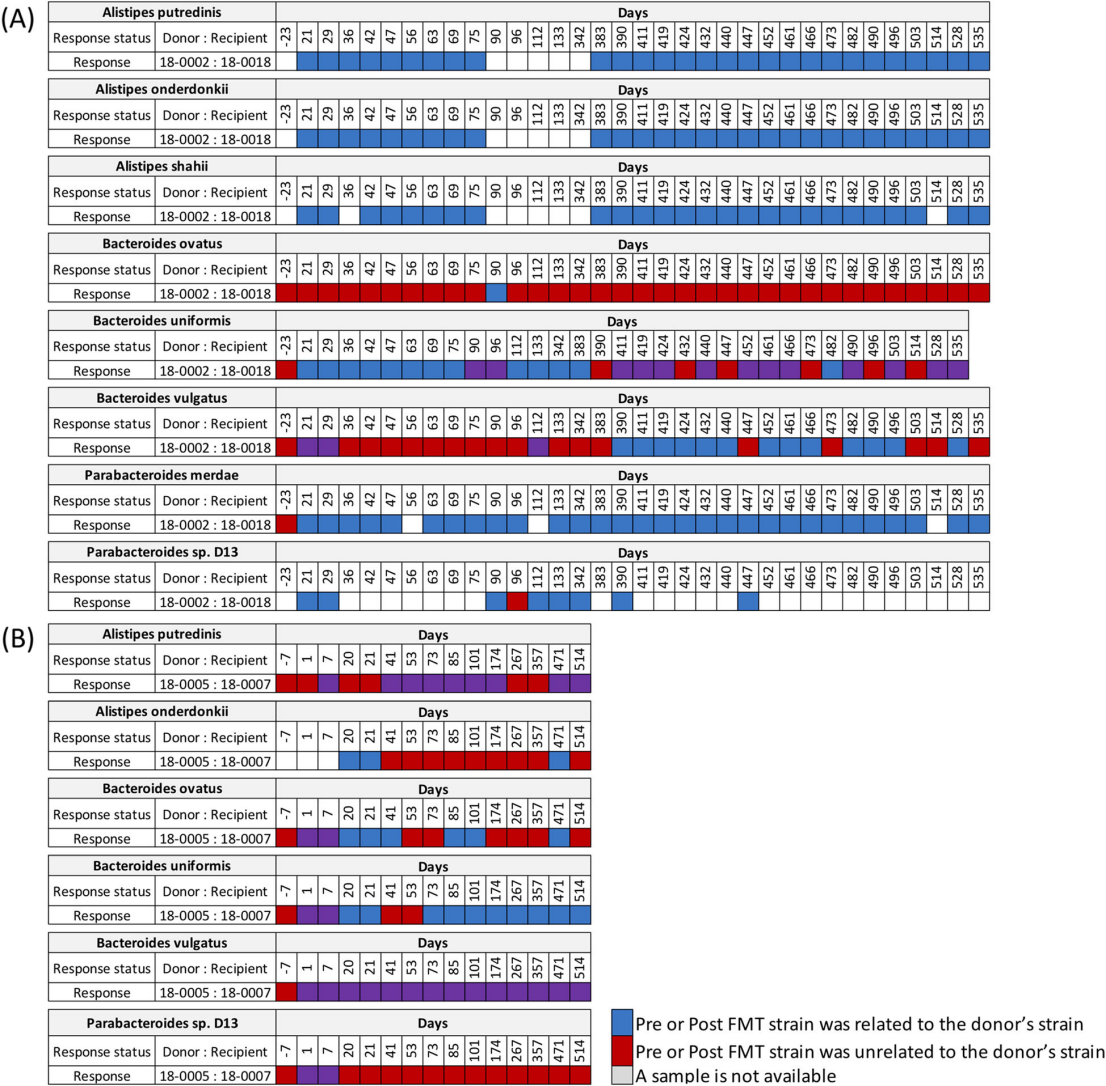
Longitudinal analysis of donor strains and recipient strains in recipients post-FMT. The WSS scores were determined by comparing the donor’s sample to every available paired recipient’s sample. In addition, the recipient’s pre-FMT sample was also compared with the same recipient’s post-FMT samples to determine strain relatedness. Only donor-recipient pairs that responded to anti-PD-1 IT were included for this analysis. (**A**) a donor of 18 − 0002 was used to multiple recipients (19–0023, 18–0018) for FMT; and (**B**) donor of 18 − 0005 was used to multiple recipients (19 − 0001, 18 − 0007). Only two donor-recipient (18 − 0002 vs. 18–0018 and 18 − 0005 vs. 18 − 0007) of four pairs were responded to the anti-PD-1 IT, and the WSS scores for all pairs used in this analysis are provided in Table S3. All samples used for this analysis are listed in Table S1. The WSS scores per each donor-recipient pair were grouped into different color boxes (see the figure key). Major species found in the fecal sample and common ones found in the two different data sets (Baruch et al. and Davar et al.) are included in this figure. The remaining species found in this data set are displayed in Fig. S4. The white boxes indicate the microbial strains that we were unable to reliably determine relatedness due to the any sample in pairs not satisfying the criteria of WSS analysis (minimum coverage > 30 % and average depth > 3.5)

To further examine strain relatedness among each recipient’s longitudinal samples, we have conducted WSS analysis on two pairs (18 − 0002 vs. 18–0018 and 18 − 0005 vs. 18 − 0007) in which IT was enhanced after FMT. To do this, we compared each recipient’s pre-FMT sample with available longitudinal post-FMT samples. Here we found that for the 18 − 0002 transplant into 18–0018 that donor strains of *A. putredinis, A. onderdonkii*, *(A) shahii*, and *P. merdae* were dominant in the recipient at all times up to 535 days post-transplant. However, in the case of *(B) ovatus*, we found that the donor strain was present in only the sample collected at 90 days. Interestingly, we found that there were pre-FMT-related strains present, including *B. uniformis*, *B. vulgatus*, and *B. stercoris* for the 18 − 0002 vs. 18–0018 pair (Fig. 4 A and Fig. S4A). In the 18 − 0005 FMT into 18 − 0007, we also found a mosaic pattern of donor and recipient dominance in the feces up to 514 days post-transplant (Fig. 4B and Fig. S4B). The results from these longitudinal analyses highlight the complexities of the FMT where at some times in the same individual the donor strains post-FMT dominate in the feces (blue boxes in Fig. 4 and Fig. S4) or recipient strains pre-FMT strain were dominant (purple boxes in Fig. 4 and Fig. S4). Finally, we also found dominant strains that were not related to either donor or recipient as determined from WSS scores below the cutoff (red boxes in Fig. 4 and Fig. S4).

### Strain-tracking to analyze the microbes in the mucosa of the intestinal epithelium of the colon

**Fig. 5:**
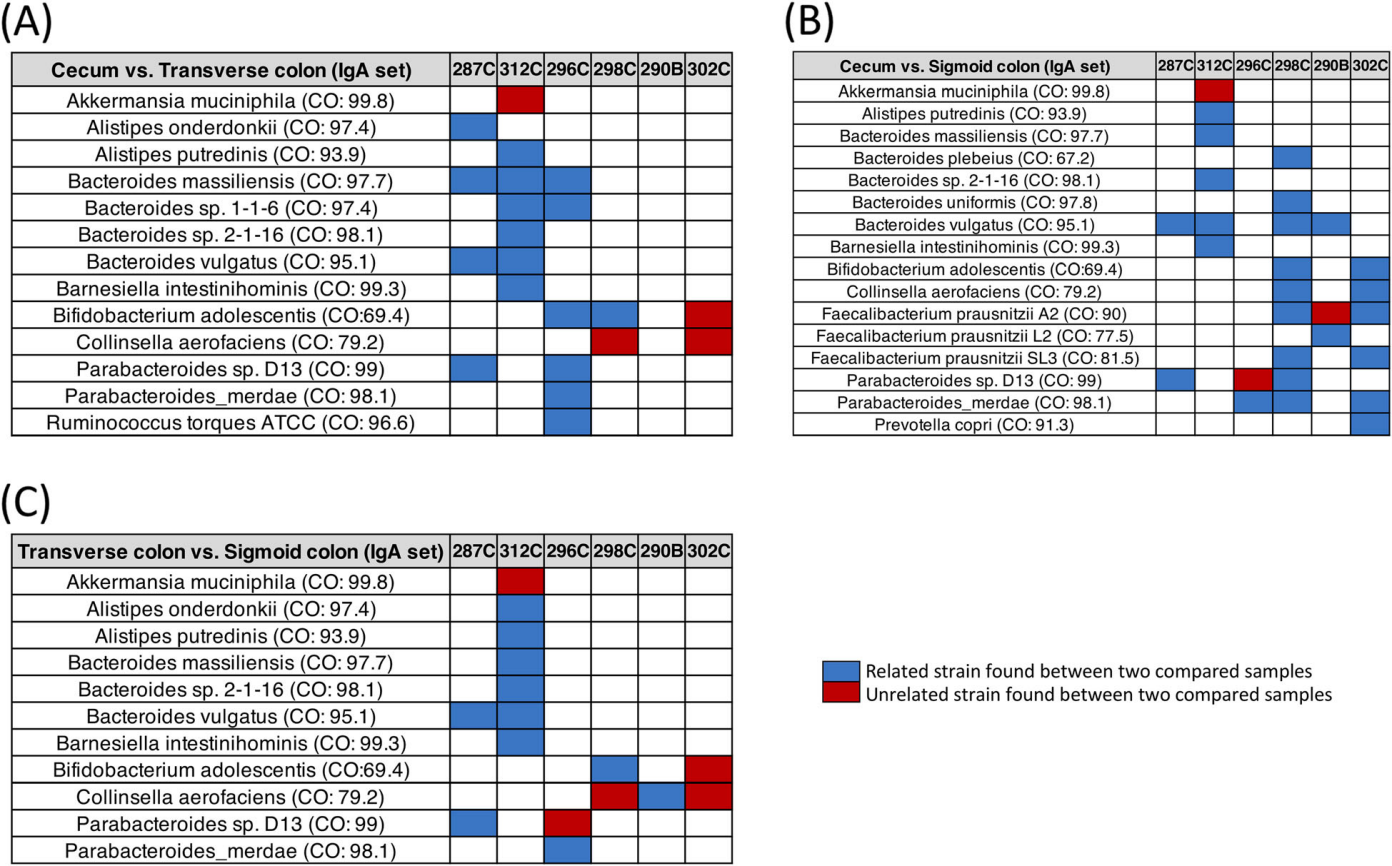
The relationship between microbial strains in the colon. Samples that were able to provide WSS scores for each species were selected to compare the WSS scores between (**A**) cecum vs. transverse colon; (**B**) cecum vs. sigmoid colon; and (**C**) transverse colon vs. sigmoid colon from IgA data sets. The summarized WSS scores were grouped into different color boxes (see the figure key). Each column in the table matches the label shown in Table S1. WSS results from analyzed IgG data sets are shown in Fig. S5, and WSS scores for all identified species are provided in Table S4. CO designates the WSS cut-off values for relatedness (Table S5). The white boxes indicate the microbial strains that we were unable to determine relatedness due to the any sample in pairs not satisfying the criteria of WSS analysis (minimum coverage > 30 % and average depth > 3.5)

There is ample evidence for the interaction of gut-associated microbes with the immune system [17, 18]. In a recent study, James et al. analyzed the microbiota found associated with the mucosa of the intestinal epithelium of the normal human cecum, transverse colon, and sigmoid colon to gain insights into the microbe interaction with the human immune system [16]. Microbes within these regions of the colon are mainly coated with immunoglobulin A (IgA) and some immunoglobulin G (IgG) [16, 19, 20]. This feature was used to isolate microbe DNA from colon segment samples of 6 individuals were used for metagenomic DNA sequencing [16]. From this data set, we used the WSS to compare the relatedness between the microbes from the colon with transverse colon and sigmoid colon segments of the 6 different individuals (Fig. 5). We found there was considerable strain sharing (relatedness) between the cecum-transverse colon and cecum-sigmoid colon for *Bacteroides spp*. (no unrelated strains) and *Parabacteroides spp.* (one unrelated strain) (Fig. 5 and Fig. S5). From our previous studies, these microbes represent the abundant species that are routinely identified in strain-tracking used to determine the relatedness between different fecal samples [11, 21]. Thus, these results demonstrate strain sharing occurs for commensal microbial strains in the intestinal epithelium throughout the entire length of the normal colon encompassing the cecum, transverse colon, and sigmoid colon, and suggests a large community structure that extends throughout the entire colon that could interact with the immune system and impact the capacity of the donor microbes derived from FMT to facilitate the response to IT.

## Discussion

In this study, we have utilized strain-tracking analysis to investigate the inter-individual variability seen for FMT to respond to IT. Strain-tracking analysis using two recent data sets demonstrates the presence of donor microbes in the recipient does not correlate with the response to IT. Further support that the link between donor microbes in the feces of recipients and response to IT is incongruent comes from the analysis of longitudinal fecal samples from recipient’s post-FMT that revealed an ongoing competition over time between the donor and recipient microbial strains for fecal dominance. Using a separate data set, we also determined that the microbial strains in the intestinal mucosa of the cecum, transverse, and sigmoid colon are shared, indicating an extensive reservoir for the recipient microbial strains to interact with the immune system. The results of our strain-tracking analysis provide evidence that the conditions used for transplant of donor microbes into existing microbial communities of the recipient do not ensure comprehensive replacement of the recipient microbes with donor and new insights to explain the inter-individual variability of FMT to enhance IT.

The number of microbes in the cecum is estimated to be approximately 10^8^ CFU per gram [22]. Both Baruch et al. and Davar et al. reported that the FMT used in their studies was administered by a colonoscope into the cecum [5, 6]. Although the number of microbes in the donor might be similar at the time of FMT, the environment of the colon is conducive to microbes of the gastrointestinal tract to increase in numbers 10,000-fold during the passage through the colon to the feces, where the number of microbes can be approximately 10^12^ CFU per gram [22]. Thus, the transplanted donor microbes would be expected to compete with the endogenous recipient samples for amplification on the feces. From our analysis of the fecal strains after early transplant, we found in some FMT the donor microbes did outcompete for the recipient microbes in the feces, while in other FMT the recipient microbes outcompeted the transplanted donor microbes and remained dominant in the feces following FMT. For the Baruch et al. study, this interpretation was complicated because of the additional sites in the colon that received FMT for the first dose and the additional FMT given to the patients (via pill) at three time points during the study at 5–9 days before collecting the fecal sample. Even with these extra doses of donor microbes though, we did not see a complete dominance of donor microbes in the recipient feces over time that correlated with the success of IT. For Davar et al., where only one FMT was given, we found separate examples where the donor microbes were the dominant fecal microbes, where a complex community of donor and recipient fecal microbial communities was established, or where the donor microbes disappeared resulting in the continued dominance of the recipient microbes in the feces [6] (Fig. 4 and Fig. S4). The three different sets of results are consistent with a previous study by Li et al. who found mixtures of donor and recipient microbes after a lean donor’s feces was transplanted into an obese recipient [23]. Collectively, the results from both studies support the concept that the ratios of the donor and recipient strains in feces can vary over time following FMT. Most probably, these changes reflect differences in competition for growth in feces that can vary as a result of changing environmental conditions, including diet [24].

One of the striking results of our strain-tracking analysis was that we identified one patient from Davar et al. following FMT (19 − 0010) that responded to IT without having donor’s (18 − 0008) strain in the feces in the recipient post-FMT [6]. This result, coupled with those in which we found the presence of donor microbes in recipients that did not respond, points to the possibility that the key to response to IT might depend on whether the donor microbes had colonized the GI epithelium. Previous studies have shown that the interaction with the host immune system occurs in the niches of the intestinal epithelium [16, 19, 20, 25]. As we have shown in our analysis, the microbes in this mucus layer from the intestinal epithelium share the same strains thorough out the cecum, transverse colon, and sigmoid colon (Fig. 5 and Fig. S5). These strains could serve as potential reservoirs for repopulation of the microbial community following disruptions. Indeed, we have shown from an analysis of microbial strains before and after a standard bowel wash that the strain profile recovered quickly, indicating that the microbes are within the intestinal epithelium niches are not eliminated by removal of the mucosa by the bowel wash [26]. More importantly, these strains throughout the intestinal epithelium could also provide a vast number of interaction sites between the microbial community and the host immune system that would be necessary for the response to IT.

We acknowledge a limitation with our analysis that we did not directly analyze the patient’s mucosal tissue in our study for microbial colonization of the intestinal mucus following FMT. Given the invasiveness of the procedure to obtain intestinal mucus from patients at varying times post- FMT, it would be unrealistic to accomplish these studies in patients undergoing cancer therapy. An alternative might be to enhance the possibility that donor microbes from an FMT access the intestinal mucosa might be to modify the FMT procedure. In our previous study, we found FMT in patients with recurrent *C. difficile* resulted in the donor microbe colonization that remained stable for up to two years, while in another study Smillie et al. found donor microbes in the recipients post-FMT for months after FMT [11]. In both of these studies, the patients had undergone antibiotic treatment that had nearly eliminated the recipient commensal microbial community prior to the FMT [11, 27, 28]. Collectively, the results from these previous studies, when taken in context with the results from new our analysis of the microbe strain conservation throughout the colon, suggests that antibiotics might be used to reduce the entire recipient microbial community prior to FMT [28]. However, as we have shown using data from a previous study by Palleja et al. that disruption of the GI microbial strain community occurred following administration of the last resort cocktail of 3 antibiotics was individual specific, indicating potentially a personalized approach would be needed for antibiotic depletion of the intestinal niches to reduce inter-individual variation [21]. Following the cessation of antibiotics, we would expect that the greater access for the donor microbes after FMT to intestinal niches would manifest in the patient as stable and dominant donor microbe strains that would provide greater opportunities for essential interactions with the host immune system necessary for enhanced effectiveness of IT.

## Conclusions

In this study, we demonstrated using strain-tracking analysis individual variation with the respect to the presence of fecal dominant donor microbes in the recipient following FMT that did not correlate with the response to anti-PD-1 immunotherapy. To help to explain this result, we show conserved microbial strains throughout the colon that could limit the opportunities for colonization by the donor microbes following FMT. Our study then supports that to reduce the inter-individual variation in the colonization of recipient’s post-FMT it might be necessary to first clear the recipient microbe niches to provide opportunities for interactions with the host immune system. Finally, additional studies will be needed to delineate the immune system function variation between individuals to fully understand the differences in the effectiveness of IT for melanoma and potentially other cancers.

## Materials and methods

### Data sets

We used publicly available data sets, James et al. [16], Davar et al. [6], and Baruch et al. [5] to conduct strain-tracking analysis. For James et al., we selected 6 individuals mucosal tissue samples (a total of 36 samples) that were collected from their colon segments (cecum, transverse colon, and sigmoid colon). The collected microbiome samples were stained, sorted for IgG^+^IgA^+^ or IgG^−^IgA^+^, sequenced, and then deposited by James et al. Further detailed sample information has been reported by James et al. [16]. For Davar et al., fecal samples from 22 individuals (7 donors and 15 recipients) were collected. For each donor, multiple samples collected at various time points were merged into a single sample to run the analysis. For each recipient, one sample was sequenced as pre-FMT (7 to 21 days prior FMT) and longitudinal samples were sequenced as post-FMT (collected weekly for 12 weeks and then every 3 weeks, if available) [6]. We selected a total of 15 donor-recipient pairs to run the analysis. For Baruch et al., fecal samples from 12 individuals (2 donors and 10 recipients) were collected. For each recipient, one sample was sequenced when colonoscopy-based FMT was conducted (0 day) and longitudinal samples were sequenced as post anti-PD-1 IT treatment (7, 31, and 65 day). All data sets used in this study were summarized in Table S1.

### DNA sequence reads and processing

A total of 3,350,868,513 metagenomic sequencing reads were downloaded from all data sets; 357,236,853 from James et al., 2,256,240,779 from Davar et al., and 737,390,881 from Baruch et al. (Table S1). Sequence reads were then filtered to remove adapters, low-quality reads (sliding window of 50 bases having a QScore < 20) and short sequences (length < 50) using Trimmomatic [29]. Host genome sequences were also filtered by mapping sequence reads to hg19 human reference genome using bowtie2, with default parameters [30]. A quality-filtered sequence reads from both data sets were then used for the downstream analyses.

### WSS Strain-tracking analysis

Our WSS strain-tracking analysis was conducted on all data sets and the full details of the WSS analysis procedure and comparison with other strain-tracking methods can be found in our previously published papers [11, 21, 26, 31–33].

For strain-tracking analyses in individuals from James et al. data set using WSS, each cecum sample was separately compared with transverse colon sample or sigmoid colon sample. Similarly, each transverse sample was separately compared with the cecum sample or sigmoid colon sample. To determine related strain pair for each sample pair (i.e. related strain pair between 1) cecum vs. transverse colon, 2) cecum vs. sigmoid colon and 3) transverse colon vs. sigmoid colon), a WSS score for each species was compared against each species’ cut-off value (related strain pair: WSS score > cut-off; unrelated strain pair: WSS score < cut-off). For Davar et al., each donor sample was compared with the paired recipient’s pre- and post-FMT samples to determine donor’s related strain in the recipient’s samples. The paired relationships between donors and recipients are included in Table S1. For Baruch et al., each donor sample was compared with the paired recipient’s colonoscopy-based FMT (0 day) and post-FMT samples (7, 31, and 65 day) samples to investigate donor’s related strain in the recipient’s samples. The FMT relationships between donors and recipients are shown in Table S1. From all analyses, species that did not have an established cut-off value were excluded for pairwise comparison. Previously established cut-off values for each species are listed in Table S5. All WSS scores found in both data sets are shown in Tables S3, S4 and S5.

## Supplementary Information

**Additional file 1: Figure S1.** Other species’ summarized WSS scores from Baruch et al. WSS analysis of the sample pairs used for this figure is provided in Fig. 1. All samples used for this analysis were listed in Table S1. The summarized WSS scores from the species that did not include in Fig. 1 were grouped into different color boxes (see the figure key). WSS scores for all pairwise comparisons are provided in Table S2.

**Additional file 2: Figure S2.** Other species’ summarized WSS scores from donors with having a single recipient. WSS analysis of the sample pairs used for this figure is provided in Fig. 2. All samples used for the analysis were listed in Table S1. The summarized WSS scores from the species that did not include in Fig. 2 were grouped into different color boxes (see the figure key). WSS scores for all pairwise comparisons are provided in Table S3.

**Additional file 3: Figure S3.** Other species’ summarized WSS scores from donors with having multiple recipients. WSS analysis of the sample pairs used for this figure is provided in Fig. 3. See Table S1 for detailed sample information. The summarized WSS scores from species that did not include in Fig. 3 were grouped into different color boxes (see the figure key). WSS scores from all pairwise comparisons are provided in Table S3.

**Additional file 4: Figure S4.** Other species’ summarized WSS scores from the longitudinal analysis. WSS analysis of the sample pairs used for this figure is provided in Fig. 4. (A) donor of 18 − 0002 was used to recipients (18–0018) for FMT; and (B) donor of 18 − 0005 was used to the recipient (18 − 0007). Summarized WSS scores for all pairs used in this analysis are provided in Table S3. All samples that used for this analysis are listed in Table S1. The summarized WSS scores from the species that were not included in Fig. 4 were grouped into different color boxes (see the figure key).

A**dditional file 5: Figure S5.** WSS scores between colon segments from IgG data set. Samples that were able to provide WSS scores for each species were selected to compare the WSS scores between (A) cecum vs. transverse colon; (B) cecum vs. sigmoid colon; and (C) transverse colon vs. sigmoid colon from IgG data sets. The summarized WSS scores were grouped into different color boxes (see the figure key). Each column in the table matches the label shown in Table S1. WSS scores for all identified species are provided in Table S4.

**Additional file 6: Table S1.** Sample and sequence read information. The original sequence files were sequenced and deposited by (A) James et al., (B) Davar et al., and (C) Baruch et al. The table represents the sample information along with sequence read information.

**Additional file 7: Table S2.** WSS scores for Baruch et al. All pairwise comparisons were conducted between a donor-recipient paired sample from Baruch et al. The resultant scores (%) are shown as a numerical value. *CO indicates the cut-off value for each species

**Additional file 8: Table S3.** WSS scores for Davar et al. All pairwise comparisons were conducted between donor-recipient paired samples. The resultant scores (%) are shown as a numerical value. *CO indicates the cut-off value for each species.

**Additional file 9: Table S4.** WSS scores for James et al. All pairwise comparisons were conducted among colon segment samples. The resultant WSS scores (%) are shown as a numerical value under the ‘WSS’ column.

**Additional file 10: Table S5.** Boundary cut-off values. A list of WSS cut-off values is provided for each species. These values were previously established based on the Human Microbiome Project (HMP) using our classifier and can be used to assess the relatedness of the strains in two separate samples in the same individual (Kumar et al., 2017).

## Data Availability

The original sequence data sets used in this study were downloaded from the European Nucleotide Archive (ENA) under accession number ERP115622 for James et al. and from the BioProject under accession numbers PRJNA672867 for Davar et al., and PRJNA678737 for Baruch et al., respectively. The datasets supporting the results of this study are included within the main text and Additional files. All codes implemented in the WSS are available at https://github.com/hkoo87/mgSNP_2.

## Abbreviations

**FMT**: Fecal Microbe Transplantation.
**IT**: ImmunoTherapy.
**SNV**: Single Nucleotide Variant.
**WSS**: Window-based Single nucleotide variant Similarity.
**HMP**: Human Microbiome Project.
**GI**: Gastrointestinal.
