# Supplementary material for: Incongruence between dominant commensal donor microbes in recipient feces post fecal transplant and response to anti-PD-1 immunotherapy
Source: BMC Microbiol. 2021 Sep 20;21:251. doi: 10.1186/s12866-021-02312-0 (PMC8454007; doi:10.1186/s12866-021-02312-0)
Supplement: Supplementary file 1 — Additional file 1: Figure S1. Other species’ summarized WSS scores from Baruch et al. WSS analysis of the sample pairs used for this figure is provided in Fig. 1. All samples used for this analysis were listed in Table S1. The summarized WSS scores from the species that did not include in Fig. 1 were grouped into different color boxes (see the figure key). WSS scores for all pairwise comparisons are provided in Table S2. [file 12866_2021_2312_MOESM1_ESM.pdf]

Fig. S1

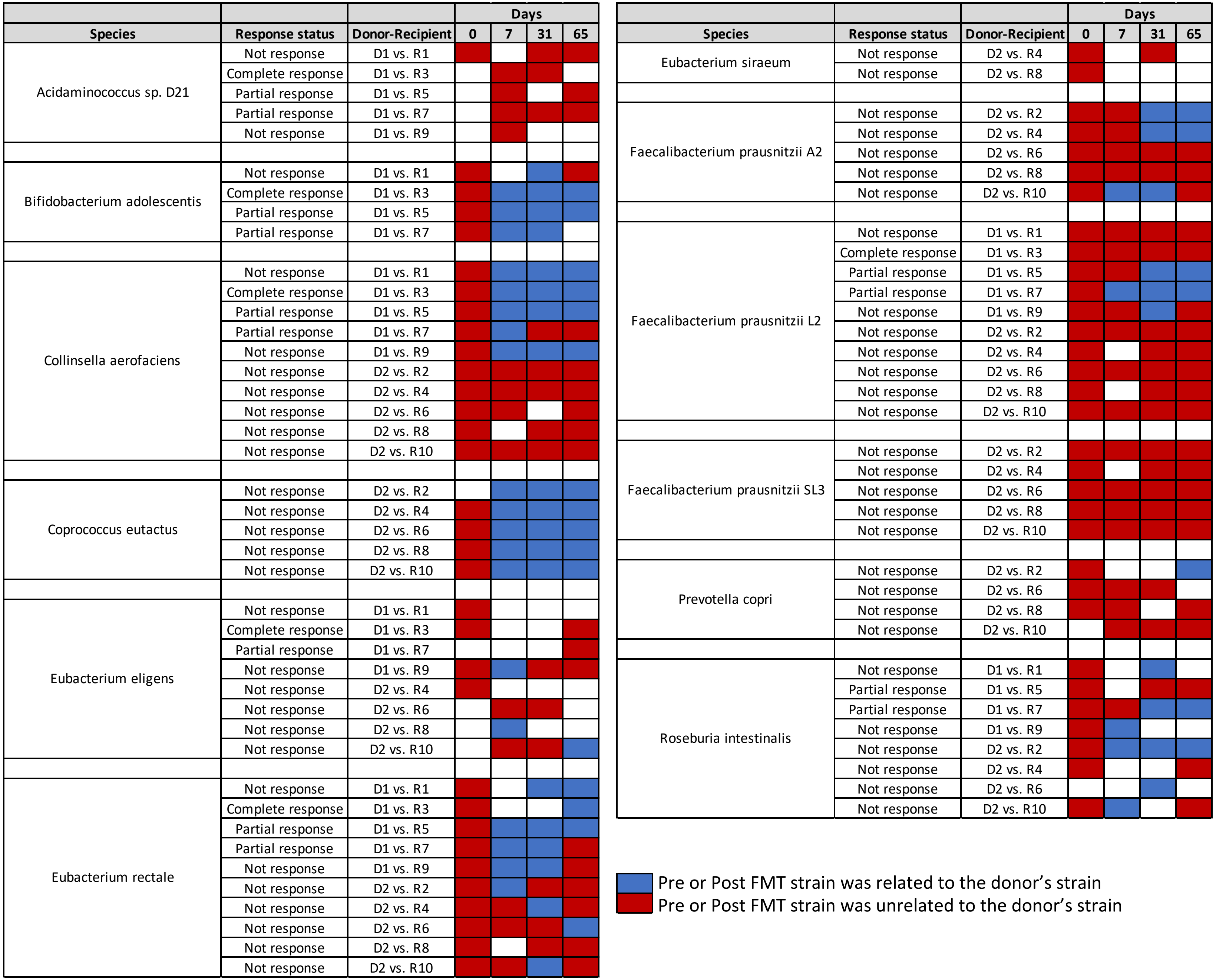
Species
Response status
Donor-Recipient
Days
0
7
31
65
Acidaminococcus sp. D21
Not response D1 vs. R1
Complete response D1 vs. R3
Partial response D1 vs. R5
Partial response D1 vs. R7
Not response D1 vs. R9
Bifidobacterium adolescentis
Not response D1 vs. R1
Complete response D1 vs. R3
Partial response D1 vs. R5
Partial response D1 vs. R7
Collinsella aerofaciens
Not response D1 vs. R1
Complete response D1 vs. R3
Partial response D1 vs. R5
Partial response D1 vs. R7
Not response D1 vs. R9
Not response D2 vs. R2
Not response D2 vs. R4
Not response D2 vs. R6
Not response D2 vs. R8
Not response D2 vs. R10
Coprococcus eutactus
Not response D2 vs. R2
Not response D2 vs. R4
Not response D2 vs. R6
Not response D2 vs. R8
Not response D2 vs. R10
Eubacterium eligens
Not response D1 vs. R1
Complete response D1 vs. R3
Partial response D1 vs. R7
Not response D1 vs. R9
Not response D2 vs. R4
Not response D2 vs. R6
Not response D2 vs. R8
Not response D2 vs. R10
Eubacterium rectale
Not response D1 vs. R1
Complete response D1 vs. R3
Partial response D1 vs. R5
Partial response D1 vs. R7
Not response D1 vs. R9
Not response D2 vs. R2
Not response D2 vs. R4
Not response D2 vs. R6
Not response D2 vs. R8
Not response D2 vs. R10
Eubacterium siraeum
Not response D2 vs. R4
Not response D2 vs. R8
Faecalibacterium prausnitzii A2
Not response D2 vs. R2
Not response D2 vs. R4
Not response D2 vs. R6
Not response D2 vs. R8
Not response D2 vs. R10
Faecalibacterium prausnitzii L2
Not response D1 vs. R1
Complete response D1 vs. R3
Partial response D1 vs. R5
Partial response D1 vs. R7
Not response D1 vs. R9
Not response D2 vs. R2
Not response D2 vs. R4
Not response D2 vs. R6
Not response D2 vs. R8
Not response D2 vs. R10
Faecalibacterium prausnitzii SL3
Not response D2 vs. R2
Not response D2 vs. R4
Not response D2 vs. R6
Not response D2 vs. R8
Not response D2 vs. R10
Prevotella copri
Not response D2 vs. R2
Not response D2 vs. R6
Not response D2 vs. R8
Not response D2 vs. R10
Roseburia intestinalis
Not response D1 vs. R1
Partial response D1 vs. R5
Partial response D1 vs. R7
Not response D1 vs. R9
Not response D2 vs. R2
Not response D2 vs. R4
Not response D2 vs. R6
Not response D2 vs. R10
Pre or Post FMT strain was related to the donor’s strain
Pre or Post FMT strain was unrelated to the donor’s strain
