# Supplementary material for: Incongruence between dominant commensal donor microbes in recipient feces post fecal transplant and response to anti-PD-1 immunotherapy
Source: BMC Microbiol. 2021 Sep 20;21:251. doi: 10.1186/s12866-021-02312-0 (PMC8454007; doi:10.1186/s12866-021-02312-0)
Supplement: Supplementary file 2 — Additional file 2: Figure S2. Other species’ summarized WSS scores from donors with having a single recipient. WSS analysis of the sample pairs used for this figure is provided in Fig. 2. All samples used for the analysis were listed in Table S1. The summarized WSS scores from the species that did not include in Fig. 2 were grouped into different color boxes (see the figure key). WSS scores for all pairwise comparisons are provided in Table S3. [file 12866_2021_2312_MOESM2_ESM.pdf]

Fig. S2

Bacteroides sp. 1-1-6		Days													
Response status	Donor : Recipient	-21	12	19	27	33	40	47	55	61	68	75	83	90	103
Not response	18-0031 : 19-0013														

Bacteroides sp. 2-1-16		Days													
Response status	Donor : Recipient	-21	12	19	27	33	40	47	55	61	68	75	83	90	103
Not response	18-0031 : 19-0013														

Bacteroides stercoris		Days																											
Response status	Donor : Recipient	-7	8	16	21	29	42	50	57	64	71	78	85	92	99	106	114	120	127	134	141	148	156	162	169	176	183	190	211
Response	18-0006 : 19-0002																												

Bacteroides cellulosilyticus		Days													
Response status	Donor : Recipient	-21	12	19	27	33	40	47	55	61	68	75	83	90	103
Not response	18-0031 : 19-0013														

Pre or Post FMT strain was related to the donor's strain

Pre or Post FMT strain was unrelated to the donor's strain
