# Supplementary material for: Incongruence between dominant commensal donor microbes in recipient feces post fecal transplant and response to anti-PD-1 immunotherapy
Source: BMC Microbiol. 2021 Sep 20;21:251. doi: 10.1186/s12866-021-02312-0 (PMC8454007; doi:10.1186/s12866-021-02312-0)
Supplement: Supplementary file 3 — Additional file 3: Figure S3. Other species’ summarized WSS scores from donors with having multiple recipients. WSS analysis of the sample pairs used for this figure is provided in Fig. 3. See Table S1 for detailed sample information. The summarized WSS scores from species that did not include in Fig. 3 were grouped into different color boxes (see the figure key). WSS scores from all pairwise comparisons are provided in Table S3. [file 12866_2021_2312_MOESM3_ESM.pdf]

Fig. S3

**Bacteroides sp. 2-1-16**		**Days**																		
Response status	Donor : Recipient	-22	-9	7	13	14	20	22	28	34	41	48	56	62	69	77	83	92	113	118
Response	18-0014 : 19-0024																			
Response	18-0014 : 18-0032																			

**Bacteroides sp. 1-1-6**		**Days**																										
Response status	Donor : Recipient	-28	-22	-9	7	13	14	20	21	22	28	29	34	35	41	45	48	56	57	62	67	69	77	83	92	113	118	118
Response	18-0014 : 19-0024																											
Response	18-0014 : 18-0032																											
Not response	18-0014 : 19-0007																											

Pre or Post FMT strain was related to the donor’s strain
Pre or Post FMT strain was unrelated to the donor’s strain
A sample is not available
