# Supplementary material for: Incongruence between dominant commensal donor microbes in recipient feces post fecal transplant and response to anti-PD-1 immunotherapy
Source: BMC Microbiol. 2021 Sep 20;21:251. doi: 10.1186/s12866-021-02312-0 (PMC8454007; doi:10.1186/s12866-021-02312-0)
Supplement: Supplementary file 4 — Additional file 4: Figure S4. Other species’ summarized WSS scores from the longitudinal analysis. WSS analysis of the sample pairs used for this figure is provided in Fig. 4. (A) donor of 18 − 0002 was used to recipients (18–0018) for FMT; and (B) donor of 18 − 0005 was used to the recipient (18 − 0007). Summarized WSS scores for all pairs used in this analysis are provided in Table S3. All samples that used for this analysis are listed in Table S1. The summarized WSS scores from the species that were not included in Fig. 4 were grouped into different color boxes (see the figure key). [file 12866_2021_2312_MOESM4_ESM.pdf]

Fig. S4

(A)

Bacteroides stercoris		Days																																
Response status	Donor : Recipient	-23	21	29	36	42	47	56	63	69	75	90	96	112	133	342	383	390	411	419	424	432	440	447	452	461	466	473	482	490	496	503	514	528	535
Response	18-0002 : 18-0018																																		

Bacteroides massiliensis		Days																																
Response status	Donor : Recipient	-23	21	29	36	42	47	56	63	69	75	90	96	112	133	342	383	390	411	419	424	432	440	447	452	461	466	473	482	490	496	503	514	528	535
Response	18-0002 : 18-0018																																		

Bacteroides sp. 1-1-6		Days																																
Response status	Donor : Recipient	-23	21	29	36	42	47	56	63	69	75	90	96	112	133	342	383	390	411	419	424	432	440	447	452	461	466	473	482	490	496	503	514	528	535
Response	18-0002 : 18-0018																																		

(B)

Bacteroides sp. 1-1-6		Days														
Response status	Donor : Recipient	-7	1	7	20	21	41	53	73	85	101	174	267	357	471	514
Response	18-0005 : 18-0007															

Bacteroides cellulosilyticus		Days														
Response status	Donor : Recipient	-7	1	7	20	21	41	53	73	85	101	174	267	357	471	514
Response	18-0005 : 18-0007															

Bacteroides sp. 2-1-16		Days														
Response status	Donor : Recipient	-7	1	7	20	21	41	53	73	85	101	174	267	357	471	514
Response	18-0005 : 18-0007															

Pre or Post FMT strain was related to the donor’s strain
Pre or Post FMT strain was unrelated to the donor’s strain
Post FMT strain was related to the recipient’s pre FMT strain
