# Supplementary material for: Incongruence between dominant commensal donor microbes in recipient feces post fecal transplant and response to anti-PD-1 immunotherapy
Source: BMC Microbiol. 2021 Sep 20;21:251. doi: 10.1186/s12866-021-02312-0 (PMC8454007; doi:10.1186/s12866-021-02312-0)
Supplement: Supplementary file 5 — Additional file 5: Figure S5. WSS scores between colon segments from IgG data set. Samples that were able to provide WSS scores for each species were selected to compare the WSS scores between (A) cecum vs. transverse colon; (B) cecum vs. sigmoid colon; and (C) transverse colon vs. sigmoid colon from IgG data sets. The summarized WSS scores were grouped into different color boxes (see the figure key). Each column in the table matches the label shown in Table S1. WSS scores for all identified species are provided in Table S4. [file 12866_2021_2312_MOESM5_ESM.pdf]

Fig. S5

(A)

Cecum vs. Transverse colon (IgG set)	287C	312C	296C	298C	290B	302C
Akkermansia muciniphila (CO: 99.8)						
Alistipes onderdonkii (CO: 97.4)						
Bacteroides massiliensis (CO: 97.7)						
Bacteroides sp. 1-1-6 (CO: 97.4)						
Bacteroides sp. 2-1-16 (CO: 98.1)						
Bacteroides vulgatus (CO: 95.1)						
Barnesiella intestinihominis (CO: 99.3)						
Bifidobacterium adolescentis (CO:69.4)						
Collinsella aerofaciens (CO: 79.2)						
Parabacteroides sp. D13 (CO: 99)						
Parabacteroides_merdae (CO: 98.1)						
Ruminococcus torques ATCC (CO: 96.6)						

(B)

Cecum vs. Sigmoid colon (IgG set)	287C	312C	296C	298C	290B	302C
Akkermansia muciniphila (CO: 99.8)						
Bacteroides plebeius (CO: 67.2)						
Bacteroides uniformis (CO: 97.8)						
Bacteroides vulgatus (CO: 95.1)						
Bifidobacterium adolescentis (CO:69.4)						
Collinsella aerofaciens (CO: 79.2)						
Faecalibacterium prausnitzii A2 (CO: 90)						
Faecalibacterium prausnitzii SL3 (CO: 81.5)						
Parabacteroides sp. D13 (CO: 99)						
Parabacteroides_merdae (CO: 98.1)						
Prevotella copri (CO: 91.3)						

(C)

Transverse colon vs. Sigmoid colon (IgG set)	287C	312C	296C	298C	290B	302C
Akkermansia muciniphila (CO: 99.8)						
Bacteroides vulgatus (CO: 95.1)						
Bifidobacterium adolescentis (CO:69.4)						
Collinsella aerofaciens (CO: 79.2)						
Parabacteroides sp. D13 (CO: 99)						
Parabacteroides_merdae (CO: 98.1)						

Related strain found between two compared samples
Unrelated strain found between two compared samples
